# Mondor’s Disease: A Rare Cause of Chest Pain in the Emergency Department

**DOI:** 10.7759/cureus.6917

**Published:** 2020-02-07

**Authors:** Brett Todd, Linnea Nierenberg, Jacob Price

**Affiliations:** 1 Emergency Medicine, Beaumont Hospital, Royal Oak, USA; 2 Emergency Medicine, Oakland University William Beaumont School of Medicine, Rochester, USA; 3 Emergency Medicine, St. Mary Mercy Hospital, Livonia, USA

**Keywords:** chest pain, thrombophlebitis, mondor's disease

## Abstract

Thrombophlebitis of a subcutaneous vein, known as Mondor’s disease, is a rare cause of chest pain and can mimic several more life-threatening diseases. Mondor’s disease can be caused by trauma, or hypercoagulable states; however, in many cases the etiology is unknown. Mondor’s disease is usually self-limited and can be managed conservatively. In this case report, we highlight a 52-year-old male patient who presented to our emergency department with chest pain caused by Mondor’s disease mimicking a pulmonary embolism. Although a rare and benign diagnosis, Mondor’s disease should be part of the differential diagnosis of chest pain and can be made on the basis of a thorough history and physical examination alone. Recognition of Mondor’s disease could reduce costs and risks of further testing for patients presenting with chest pain.

## Introduction

Chest pain presents a diagnostic challenge to the clinician with etiologies that range from life-threatening to benign [1]. Often an extensive diagnostic evaluation is performed; however, in some instances the history and physical examination alone may reveal the diagnosis. Thrombophlebitis of the subcutaneous veins of the chest wall, known as Mondor’s disease, is one such diagnosis. It is most frequently identified in the chest wall, although it has also been described in the groin, penis, and antecubital fossa [2]. Below we present a case of chest pain seen in our emergency department, mimicking a pulmonary embolism, that was ultimately diagnosed as Mondor’s disease. The patient described in this case presented with symptoms classically seen in Mondor’s disease-a hard, indurated, cord-like mass that is painful upon palpation without overlying erythema [3]. 

## Case presentation

History

A 52-year-old African American male presented to the emergency department complaining of pleuritic, sharp pain in his right chest wall that had begun three days prior to arrival. The pain had since worsened in intensity, and did not radiate. He had taken acetaminophen without relief of his symptoms. The patient denied any fever, cough, shortness of breath, headache, or rash. His review of symptoms was otherwise negative. The patient had no significant past medical history and was taking no medications. He denied any recent history of surgery or trauma, but did note weight lifting two days prior to the onset of symptoms. Family history was negative for coagulopathies and autoimmune conditions. 

Exam findings

Vital signs on arrival were blood pressure 146/97, heart rate 97, temperature 36.2°C, respiratory rate 20, and SpO_2_ 99% on room air. He was alert and oriented, and in no acute distress. Lung sounds were clear bilaterally and heart sounds were normal. A raised cord was visible crossing from the epigastrium across the right breast toward the axilla, that was tender to palpation and mobile. There was no warmth, erythema, or rash overlying the cord. Head/eyes/ears/nose/throat, abdominal, and neurological examinations were unremarkable.

Diagnostic evaluation

**Figure 1:**
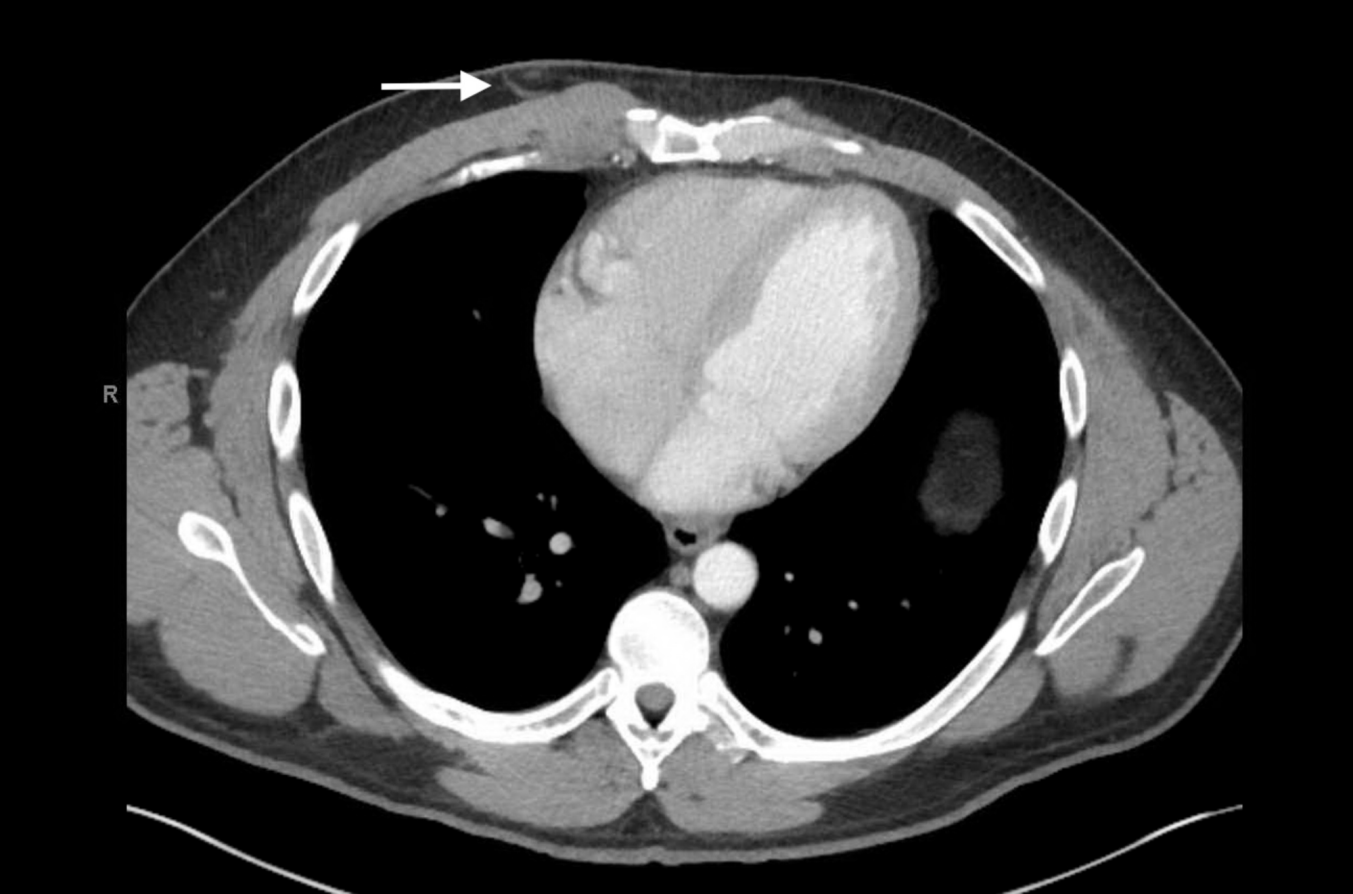
Computed tomography scan showing a subcutaneous thrombosis in the right chest wall.

Basic metabolic panel and troponin levels were normal, and a complete blood count and differential was unremarkable with platelets of 212 bil/L. Chest radiograph showed clear lung fields but did reveal remote right-sided posterior fractures of ribs five through eight. Given the pleuritic nature of the pain, a computed tomography pulmonary angiogram was ordered to rule out pulmonary embolism. As shown in Figure 1, it revealed a subtle density in the right chest wall over the area of the palpable cord, with no evidence of a pulmonary embolism. The diagnosis of superficial chest wall thrombophlebitis, or Mondor’s disease, was made. 

Patient course

The patient was treated with ibuprofen in the emergency department and discharged with a naproxen prescription. He was referred to his primary care physician for further work-up. The pain improved in the days following his emergency department visit, resolving within a week of discharge. The patient was contacted two months after his emergency department visit. He noted that he was still able to appreciate a firm mass in his chest wall with some reduction in size since it was first diagnosed. 

## Discussion

Thrombophlebitis of the subcutaneous veins, also known as Mondor’s disease, was first described in 1939 by Henry Mondor as “string phlebitis” [2]. Mondor’s disease has been associated with many inciting processes, including muscular strain, repetitive movement of the arm, trauma, inflammatory processes, surgery, biopsy, infection, and breast carcinoma; however, a specific etiology is frequently not identified [2]. Mondor’s disease may present either unilaterally or bilaterally [4]. It is reported more frequently in women than men by a ratio of 3:1 [5]. In females, Mondor's disease is most often related to breast surgery or trauma [6]. In males, penile Mondor’s is frequently associated with sexual activity [7]. Mondor’s disease can reoccur multiple times but is not usually considered a chronic condition [8].

The diagnosis of Mondor’s disease is primarily clinical, although CT and ultrasound can be utilized when the diagnosis is in question. Ultrasound can reveal non-compressibility and echogenicity [2,9,10]. Color Doppler can also reveal a lack of blood flow upon initial presentation and return of perfusion upon recanalization during the healing process [3,9]. It can be mistaken for a lymphangitis and in the case of penile Mondor’s disease, sexually transmitted infections and Peyronie’s disease should also be taken into consideration [3]. As Mondor’s disease has been associated with potentially severe etiologies such as hypercoagulability, inflammatory conditions, and breast carcinoma, consideration should be given to evaluating these conditions [2,5].

Mondor’s disease is ultimately a benign process, and thus its treatment is largely symptomatic [5,11]. Treatment with non-steroidal anti-inflammatory drugs and warm compresses are often sufficient for resolution of symptoms [12]. Recommendations for the treatment of superficial thrombophlebitis more broadly do include four weeks of low molecular weight heparin as per the American College of Chest Physicians, and while cases of such an approach to Mondor’s disease specifically do exist, treatment is typically more conservative [13]. For female patients with Mondor’s disease of the breast, topical diclofenac sodium patches have been used to inhibit prostaglandin-mediated inflammation [14]. When it is located in the axillary region, it may also be possible to manually disrupt the fibrosis with applied pressure [3]. Lastly, in cases of persistent symptoms, excision of the vein may be necessary [12]. Although biopsies were once part of the work-up of Mondor’s disease, they are no longer recommended due to its self-limited course [3,5].

## Conclusions

Thrombophlebitis of the chest wall (Mondor’s disease) is a benign cause of chest pain, which can mimic more serious etiologies. The diagnosis can be made on the basis of a thorough history and physical examination, with imaging and laboratory testing only needed to exclude more concerning diagnoses. The treatment is usually symptomatic, although low molecular weight heparin has been used in some cases. Failure to consider Mondor’s disease in the chest pain patient can result in increased cost and unnecessary testing. As such, it is crucial that practitioners keep Mondor’s disease in the differential diagnosis when approaching localized chest pain with a palpable mass.
